# Screening Potential Probiotic Characteristics of* Lactobacillus brevis* Strains In Vitro and Intervention Effect on Type I Diabetes In Vivo

**DOI:** 10.1155/2018/7356173

**Published:** 2018-09-19

**Authors:** Amro Abdelazez, Heba Abdelmotaal, Smith Etareri Evivie, Sherif Melak, Fang-Fang Jia, Mir Hassan Khoso, Zong-Tao Zhu, Lu-Ji Zhang, Rokayya Sami, Xiang-Chen Meng

**Affiliations:** ^1^Key Laboratory of Dairy Science of Ministry of Education, Northeast Agricultural University, Harbin 150030, China; ^2^Department of Dairy Microbiology, Animal Production Research Institute, Agriculture Research Centre, Dokki, Giza 12618, Egypt; ^3^Department of Microbiology, Soil, Water, Environment, and Microbiology Research Institute, Agriculture Research Center, Giza 12619, Egypt; ^4^Department of Microbiology and Biotechnology, College of Life Sciences, Northeast Agricultural University, Harbin 150030, China; ^5^Department of Animal Science, Faculty of Agricultural, University of Benin, 1154, Benin City, Edo State, Nigeria; ^6^Department of Animal Science and Biotechnology, College of Animal Science and Technology, Nanjing Agricultural University, Nanjing 210095, China; ^7^Department of Sheep and Goat, Animal Production Research Institute, Agriculture Research Centre, Dokki, Giza 12618, Egypt; ^8^Department of Food Science and Nutrition, Taif University, Taif, Al-huwayah 888, Saudi Arabia

## Abstract

Diabetes has become the third most serious threat to human health, after cancer and cardiovascular disease. Notably,* Lactobacillus brevis* is the most common species of LAB that produces *γ*-aminobutyric acid (GABA). The aim of this study is to clarify the effect of time, strain types, antibiotic concentrations, different levels of pH, and intestinal juices in aerobic or anaerobic conditions and the effect of interactions between these factors on the potential properties of KLDS 1.0727 and KLDS 1.0373, furthermore, antagonistic activity against foodborne pathogens. Moreover, another aim is to study the capability of KLDS 1.0727 and KLDS 1.0373 strains as* gad* gene carriers to express GABA that reduce the risk of type 1 diabetes in C57BL/6 mice as diabetic models. The obtained results exhibited the surprising tolerance of* Lactobacillus brevis* strains in vitro digestion models mimicking the conditions of the gastrointestinal tract, further, large antagonistic activity against foodborne pathogeneses. In vivo results displayed the significant effect on glucose level reduction, blood plasma, and histological assays of mice organs. As recommended, the use of* Lactobacillus brevis *strains should be widely shared in the market as a natural source of GABA in pharmaceutical and food applications.

## 1. Introduction

Nowadays, food plays a vital role in inhibiting diseases and ensuring health. One of the most important challenges facing customers is the safety of high-quality healthy foods and the creation of a new diet that focuses on the prevention of chronic diseases and disorders [1]. Functional food acts as beneficial compounds or foods containing microorganisms exhibiting a pivotal role in strengthening and enriching health well-being and suppressing some strict disease, for instance, obesity, diabetes, atherosclerosis, heart disease, retinopathy, kidney toxicity, atherosclerosis, hypertension, diabetic foot ulcers, and cystic fibrosis [2, 3]. The most vital components of new functional foods are called probiotics, which can increase host immunity and improve health benefits [4]. Recently, various conventional microorganisms have been used for probiotic cultures in fermented foods. Furthermore, probiotics include a wide range of pharmaceuticals or foods and special dietary applications such as juices, nutrition bars, infant formulas, and cosmetics [5].

The medical application of* Lactobacillus brevis* KB 290, isolated from “Suguki”, is a traditional Japanese pickle that plays a crucial role against people with influenza [6]. In addition, it plays a vital role in suppressing inflammation caused by nitric oxide [7]. Several studies have been conducted on several* Lactobacillus brevis* strains isolated from traditional Egyptian dairy products, which have been tested to confirm their antimicrobial effects. These results show the extreme activity of* Lactobacillus brevis* B23 among 38 species [8].* Lactobacillus brevis* is a vital biological organism to produce bioactive *γ*-aminobutyric acid biologically [9]. Formerly, *γ*-aminobutyric acid (GABA) has been attracting attention because it is the most abundant inhibitory neurotransmitter that maintains the neurotransmitter functions of the human central nervous system. Furthermore, GABA may not have the ability to penetrate the human blood-brain barrier [10].* gad* antibodies, a symptom of type 1 diabetes, can be detected before the onset of clinical disease and the identification of a subset of patients with adult autoimmune disease [11].

Currently, more than a thousand LAB strains have been sequenced and deposited in the NCBI GenBank database. Therefore, genetic assays were performed in most continuous strains in the presence or absence of* gad* operon and genes encoding glutamate decarboxylase in most of the sequenced strains. Moreover, there are not only differences between microbial species from different sources in the* gad* enzymatic* properties* but also differences between the subspecies as well. Lactobacillus different sources of* gad* subunit composition and molecular weight have a huge difference.* Lactobacillus brevis gad* has two subunits molecular weight of 57, 60 kDa [12].

*γ*-Aminobutyric acid (GABA) is a nonprotein, four-carbon, free amino acid, synthesized by the irreversible *α*-decarboxylation reaction of L-glutamic acid or its salts, a pyridoxal-50-phosphate-dependent enzyme (PLP; active vitamin B6)[13]. GABA is permitted as a dietary ingredient and its nutrients have shown antihypertensive and antidepressant activities as the two main functions of the host after oral administration [9]. However, the GABA content in a few natural plants and animal products has been further studied in the LABs because it also possesses the ability to produce GABA.

Type 1 diabetes (T1D) is an autoimmune disease characterized by selective destruction of the pancreatic beta cells [14]. Furthermore, it is a metabolic disease induced by abnormal insulin secretion by damaged islet *β* cells. That damage accrues resulting in insulin insufficiency, which can lead to life-threatening hyperglycemia that manifests clinically as weight loss, polyuria, polyphagia, and polydipsia [15]. LAB could be utilized to grab foodborne diseases, researchers used* Salmonella typhimurium*, which causes vomiting, abdominal pain, nausea, and diarrhea [16],* Listeria monocytogenes*, which causes abortion and gastrointestinal diseases [17],* Staphylococcus comets*, which is involved in harmful gastroenteritis, and* E. coli*, thereby exacerbating the syndrome and colitis [18].

The main objective of the present study was to examine the new* Lactobacillus brevis* probiotic strains that could efficiently produce high levels of bioactive *γ*-Aminobutyric Acid (GABA) by exploring* gad* gene, in addition, study potential probiotic properties in vitro where they remain in severe case of simulation gastrointestinal juice and different types of antibiotics and, moreover, antimicrobial activity against foodborne pathogens, and finally, control the development of type 1 diabetes in vivo.

## 2. Materials and Methods

### 2.1. Bacterial Strains and Cultivation Conditions

Two strains of* Lactobacillus brevis* KLDS 1.0727 and KLDS 1.0373 were frozen stored at −80°C. Further, the foodborne pathogenic microorganisms used were* Salmonella typhimurium* (ATCC® 14028-MINI-PACK™) (American Type Culture Collection),* E. coli* IQCC 10126,* Listeria monocytogenes* IQCC 22221, and* Staphylococcus aureus* IQCC 22035 (Inspection and Quarantine Culture Collection of the Chinese Academy of Inspection and Quarantine culture libraries, IQCC). All test strains were obtained from Key Laboratory of Dairy Science (KLDS), Heilongjiang Province Harbin, China.

*Lactobacillus brevis* KLDS 1.0727 and KLDS 1.0373 strains were inoculated (1% v/v) and grown in De Man Rogosa Sharpe medium (MRS Oxoid), while foodborne pathogenic bacteria were grown in Tryptic Soy Broth (TSB) (Sigma Aldrich). All the strains were cultivated at 37°C aerobically for 24 hours. The solid plates medium was cultivated for 48 hours, single colonies were picked, after examination of the pure bacteria, and they were then cultivated in an adequate liquid culture medium.

KLDS 1.0727 and KLDS 1.03737 colonies were streaked on MRS agar plate and incubated at 37°C for 24 hours under aerobic conditions. Colonies were randomly picked from the plates and subculture two or three times on fresh MRS agar plates. A single colony from the plate was transferred to MRS broth and incubated for 18 hours at 37°C. The pure KLDS 1.0727 and KLDS 1.0373 cultures were kept in MRS broth supplemented with 30% (v/v) glycerol and frozen at −80°C until further analysis.

### 2.2. Screening of* Lactobacillus brevis* KLDS 1.0727 and KLDS 1.0373 Strains for the Presence of Glutamic Acid Decarboxylase (*gad*) Gene

Genomic DNA was prepared using TIANamp Bacterial DNA Kit (Tiangen Biotech Co., Ltd, Beijing, China) and with some modification of manufacturer's extraction steps KLDS 1.0727 and KLDS 1.0373 were lysed with lysozyme DNA extracted.

### 2.3. PCR Amplification of the 16S rDNA and Sequence Analysis

PCR amplification was performed using the universal primers:

**Table:** 

Primer ID	Sequences (5′ → 3′	Reference
Lb-PTC-F	GCCAGAAACGCTCAAGAT	[19]
Lb-PTC-R	GGCTTCGTATAAGCCATACC	
Lb-OTC-F	GTGAAAGCAACTGGGAAGA	
Lb-OTC-R	GTTATGGAAAGCAGGCAAAC	
Lb-TDC-F	CGATCAAGCAGAGTCCATTAC	
Lb-TDC-R	CGGCACCCTTCTCAAATAC	

Genomic DNA was used as a template for PCR amplification. Fifty microliters of each PCR mixture contained DNA template 1.0 *μ*L, two primer pairs (ComateBio Custom Primers, Jilin, Changchun, China),* gad*-F/*gad*R (10 *μ*mol / L) 2.0 *μ*L, [19] DNA Polymerase (2.5 U / *μ*L) 0.5 *μ*L, 10× Taq Buffer 5.0 *μ*L, 2'-deoxynucleoside 5' triphosphate (dNTPs) (2.5 mM) 4.0 *μ*L, and ddH_2_O 35.5 *μ*L. Furthermore, PCR amplification was performed using the GeneAmp PCR System 9700 thermal cycler (Applied Biosystem, USA) with the thermal cycling parameters as follows: denaturation at 95°C for 5 min, 30 cycles of 95°C for 30 s, 55°C for 1.30 min and 72°C for 1.30 min, with a final extension at 72°C for 10 min. A 5-*μ*L volume of PCR product was electrophoresed on a 1% agarose gel (Gibco BRL, France) at 90 V for 1.5 hours and then stained with ethidium bromide and band patterns were visualized. Sequence similarity analysis was performed in the GenBank database using the BLAST program (http://blast.ncbi.nlm.nih.gov/Blast.cgi) (NCBI).

### 2.4. Evaluation of KLDS 1.0727 and KIDS 1.0373 In Vitro Digestion Models Mimicking the Gastrointestinal Tract

In vitro assessment has been tested for the viability of KLDS 1.0727 and KLDS 1.0373, for example, growth rate, bile salts, simulated intestinal juice (SIJ) at different pH levels under aerobic or anaerobic conditions [20], and surviving in a wide range of different antibiotics types. The antagonism of various foodborne pathogens and, furthermore, all experiments were expressed in triplicate using the MRS broth as a blank. In addition, the absorbance was measured with a wavelength of 620 nm and the data were expressed as OD.

#### 2.4.1. Standard Curve of Growth Rate and pH for KLDS 1.0727 and KLDS 1.0373 strains

KLDS 1.0727 and KLDS 1.0373 strains were inoculated (1% v/v) and grown in MRS broth at 37°C under aerobic conditions. The growth bacteria were monitored using a spectrophotometer (F-7000, Hitachi High Technologies, 155 Shenzhen, China), and pH was determined by using pH meter (MP 220, Mettler Toledo, Greifensee, Switzerland).

#### 2.4.2. Resistance of KLDS 1.0727 and KLDS 1.0373 to Different Bile Salts Concentration in MRS Incubated at 37°C

KLDS 1.0727 and KLDS 1.0373 were tested for hydrolyzed bile salt activity. The strains were cultured in MRS broth added to 0.3, 1, and 2% (w/v) of the bovine bile (Difco, DB Diagnostic System, reference 212820) and incubated at 37°C under aerobic conditions, then the tested media were obtained as a filtration solution through a 0.22 *μ*m filter (Critical Syringe Filters; Critical Process Filtration Inc). The activated strains in the MRS broth were taken without Ox gall powder as a control [21]. The following equation expressed the survival percentage of bile salts after 33 hours.

(1) $$\begin{array}{c} Bile\;\;survival\%=\frac{\log N1}{\log N0}\times 100 \end{array}$$

N1 is absorbance of cultures in MRS broth containing 0.3, 1, and 2 % bile salts (see [22]). log⁡N0 is absorbance of cultures in MRS broth without bile salts.

#### 2.4.3. Resistance of KLDS 1.0727 and KLDS 1.0373 to Different Types of Antibiotics

The microdilution protocol was used to assess the resistance of probiotic bacteria to different types of antibiotics [23] with some modifications according to EFSA Guidance [24]. The antibiotics ampicillin, chloramphenicol, erythromycin, gentamycin, kanamycin (Sigma Aldrich, St. Louis, MO), and streptomycin (MP Biomedicals, Santa Ana, CA) and the amounts of the active compound were placed into the broth media tube: ampicillin (10 *μ*g), chloramphenicol (30 *μ*g), erythromycin (15 *μ*g), gentamycin (10 *μ*g), kanamycin (30 *μ*g), and streptomycin (10 *μ*g). Cultures were inoculated in MRS broth filtered through a 0.22-*μ*m filter and incubating aerobically at 37°C overnight. Furthermore, antibiotic stock solutions were prepared according to the following formula:

(2) $$\begin{array}{c} W=\frac{1000}{P}\times V\times C \end{array}$$

where P is potency given by the manufacturer (*μ*g /mg), V is volume required (ml), C is final concentration of solution (multiples of 1000, mg/L), and W is weight of antibiotic (mg) to be dissolved in volume V (mL). The data was expressed after 24 hours as the following formula:

(3) Survival%=log⁡N1log⁡N0×100

(4) Sensitivity  of  antibiotic=100−survival%

log⁡N1 is absorbance of culture 620 nm in MRS broth with different antibiotic types and log⁡N0 is absorbance of culture 620 nm in MRS broth as a control.

#### 2.4.4. Tolerance of KLDS 1.0727 and KLDS 1.0373 to Simulated Intestinal Juice (SIJ) with Different pH under Aerobic or Anaerobic Conditions

*Lactobacillus brevis* strains are a facultative anaerobic LAB strains. Therefore, KLDS 1.0727 and KLDS 1.0373 strains were propagated in Lactobacilli MRS broth for 24 hours. Simulated intestinal juice (SIJ) (grams/100 ml) was prepared as follows: 0.1 of trypsin; 1.8 of bile salts; 1.1 of sodium bicarbonate; 0.2 of sodium chloride followed by adjusting the final pH to 2.0, 3.0, and 7.0 and sterilizing the obtained solution by filtration through 0.22 *μ*m filter [25]. Afterwards, an appropriate amount (10^9^ CFU/ mL^−1^) of the freshly prepared inoculum of KLDS 1.0727 and KLDS 1.0373 strains was inoculated into the prepared (SIJ) and incubated for 0, 3, 6, and 24 hours. Moreover, anaerobic conditions designed by anaerobic glove chamber (Sheldon Manufacturing, Inc., Shel LAB, Cornelius, OR, USA) were used with a gas mixture of (90% N_2_, 5% CO_2_, 5% H_2_). According to the comparison of these strains in the ability to survive after 24 hours,

(5) $$\begin{array}{c} Survival\%=\frac{\log N1}{\log N0}\times 100 \end{array}$$

where log⁡N1 is absorbance of culture 620 nm in MRS broth at pH 2, 3, and 7 and log⁡N0 is absorbance of culture 620 nm in MRS broth as a control.

#### 2.4.5. Screening Antagonistic Properties of KLDS 1.0727 and KLDS 1.0373 Strains

Antagonistic activities were assessed by measuring clear zones mm from the edge as described by Damaceno et al. [26]. Two single strains of KLDS 1.0727 and KLDS 1.0373 and foodborne pathogens* Salmonella typhimurium* ATCC 14028,* E. coli* IQCC 10126,* Listeria monocytogenes* IQCC 22221, and* Staphylococcus aureus* IQCC 22030 as indicator strains studied for antagonism were used. Agar slabs of 6-mm in diameter were aseptically cut off from the MRS agar overgrown with a lawn of KLDS 1.0727 and KLDS 1.0373 strains incubated for 24 hours at 37°C, under aerobic conditions and placed on plates with the Tryptic Soy Medium agar inoculated with the indicator strain (10^5^–10^6^ CFU/ mL). The plates were kept at 4°C for 3 hours to permit diffusion on the assay material and incubated at 37°C for 24 hours of incubation and then the diameters of clear zones around the agar slabs were measured. To compare the antagonistic activity of KLDS 1.0727 and KLDS 1.0373 strains against the indicator cultures, the clear zones were expressed as 14 mm, 3 points; 9–14 mm, 2 points; 1–8.9 mm, 1 point.

### 2.5. Caco-2 Cell Adhesion Assay

The ability of KLDS 1.0727 and KLDS 1.0373 strains was tested for adherence to human epithelial cells using Caco-2 cells (human colonic adenocarcinoma, ATTC HTB-37) [27]. Monolayers of Caco-2 cell line obtained from China Cell Bank, Shanghai, were grown in Dulbecco's modified Eagle's Medium (Sigma, Aldrich) high glucose supplemented with 10% (v/v) fetal bovine serum (Gibco, reference 12484-028), 1% (v/v) nonessential amino acid, and 1% (v/v) penicillin-streptomycin at 37°C in a humidified atmosphere of 95% air and 5% CO_2_. The Caco-2 cell concentration was adjusted to 5×10^5^ cell/mL and seeded into well tissue culture plates and, subsequently, incubated at 37°C in a 5% CO_2_ incubator until the Caco-2 cells attained a confluent differentiated monolayer state (15 ± 1 d). The Caco-2 monolayer was washed twice with sterile PBS to eliminate the penicillin-streptomycin. The KLDS 1.0727 and KLDS 1.0373 strains were harvested by centrifugation (10,000 g, 5 min, 4°C) and washed twice with sterile PBS. Then, density was adjusted with high-glucose DMEM without antibiotics to 10^8^ CFU/mL. Afterwards, 1 mL of each KLDS 1.0727 and KLDS 1.0373 strains suspension were added to the wells, and the plates were incubated for 2 hours at 37°C in a 5% CO_2_ atmosphere. At the end of the assay, Caco-2 cells were washed three times with sterile PBS to remove unadhered KLDS 1.0727 and KLDS 1.0373 cells.

The Caco-2 monolayers were lysed by treatment with EDTA-trypsin solution for 3–5 min at 37°C for disrupting the adherent cells. The Caco-2 lysate and the attached KLDS 1.0727 and KLDS 1.0373 cells were plated on MRS agar plate after serial dilution and counted after 24 hours of incubation at 37°C. The adhesion ability of KLDS 1.0727 and KLDS 1.0373 was determined using the following formula:

(6) $$\begin{array}{c} Adhesion\%=\frac{\log Nt}{\log N0}\times 100 \end{array}$$

where log⁡Nt is the number of KLDS 1.0727 and KLDS 1.0373 strains that adhered to the Caco-2 monolayers, and log⁡N0 is a total number of KLDS 1.0727 and KLDS 1.0373 strains added as a control blank.

Moreover, the direct microscopic examination method was used to disable the adhesion of KLDS 1.0727 and KLDS 1.0373 with Caco2 cells after (15 ± 1 d) of cultivation as described above. The culture medium was replaced with an antibiotic-free medium one day prior to the adhesion assay. Afterwards, the cells were washed twice with phosphate-buffered saline (PBS) (pH = 7.2); KLDS 1.0727 and KLDS 1.0373 strains were added to the prepared cell monolayers. After 2 hours of incubation at 37°C, all monolayers were washed 5 times with PBS to remove nonadherent bacteria. Finally, binding between* Lactobacillus brevis* strains and Caco2 cells was examined by Gram-stained phase contrast microscopy (magnification fold, 200 x). The adhered* Lactobacillus brevis* strains Caco2 cells were determined in 15 randomly selected microscopic fields.

### 2.6. In Vivo Experiments

#### 2.6.1. Animal Experiments Design and Establishment of Diabetic Model Mice

Specific pathogen-free (SPF) male mice C57BL/6 (6-8 weeks old) were purchased from Vital River Laboratory Animal Technology Co., Ltd. (Beijing, China), housed in a room under controlled environmental conditions at 23±2°C, a relative humidity of 50±20% with artificial light cycle a 12-h light/dark. Mice were raised in independent, ventilated cages and received pathogen-free food and water. The mice were acclimatized for one week of the laboratory conditions before beginning the experiments. The experimental protocol was approved by the Institutional Animal Care and Use Committee of the Northeast Agricultural University under the approved protocol number specific pathogen-free rodent management (SRM)-06.

Table 1 shows the animal experimental designed; the animals were divided into five groups within four mice per group. Afterwards, the diabetic mice were treated with high dose of streptozotocin (STZ, 180 mg/kg) (Sigma, Aldrich) freshly prepared in 50 mM sodium citrate buffer (pH 4.5) and subcutaneously injected within 10 to 15 min after dissolving for one time according to a previously described procedure [28].

The nondiabetic control group received an injection of citrate buffer only 3 days post-STZ-injection; glucose levels were measured using a glucometer (Yuwell, Jiangsu, China). The mice with glucose levels higher than ≥7 mmol/dl were considered diabetic and STZ-induced mice that had a lower glucose level were excluded.

#### 2.6.2. Weekly Determination of Glucose Level and Body Weight of Streptozotocin-Induced Diabetic Mice

Following blood glucose, blood glucose level and body weight of overnight fasting for 12 hours and 2 hours postprandial were assessed weekly. Glucose levels were measured using a glucometer and glucose expressed as mmol/ dl.

(7) Glucose  level=Postprandial  2  hours−Fasting  12  hours

#### 2.6.3. Plasma Biochemical Analyses

After feeding for four weeks, fasting blood samples were collected from the ocular vein of each group and allowed to clot at 4°C and then centrifuged at 12,000 × g for 10 min. The plasma was transferred to a new microcentrifuge tube and stored in a -80°C for biochemical measurements. The blood plasma was determined for serum lipids concentrations as triglyceride (TG); total cholesterol (CHOL); high-density lipoprotein cholesterol (HDL); low-density lipoprotein cholesterol (LDL); glucose (GLU); magnesium (Mg^+2^) which were measured. Furthermore, liver functions were evaluated by assessing serum alanine aminotransferase (ALT), aspartate transaminase (AST); AST/ALT; total bile acid (TBA); albumin (ALB); globulin (GLUB); total protein (TP). Moreover, determination of kidney functions such as uric nitrogen (BUN); creatinine (CREA); uric acid (URIC) levels was assessed as well. All test parameters were a determination by using a Beckman Coulter UniCel DxC 800 (Beckman Coulter, Miami, FL, USA) analyzer.

#### 2.6.4. Serum Insulin Determination

Mice were fed rodent chow for 4 weeks with injected daily with insulin (Sigma) using dose unit (0.5 units/ kg body weight). Insulin was diluted in acetic acid for a final injected volume of 100 *μ*l. Insulin sensitivity was evaluated essentially as described by Surwit et al., [29]. Blood serum insulin was detected by insulin determination ELISA kits (Meimian Biotech Co., Ltd., Yancheng, China). The experimental process was according to the manufacturer's instructions of insulin kits.

#### 2.6.5. Histological Evaluation

The experimental procedures used for routine histological examination of organs in mice tissue were previously described [30]. Briefly, the mice organs (liver, pancreas, kidney, and spleen) were removed and washed by phosphate buffer (pH 7.2) and then fixed in 10 % neutral formalin, followed by dehydrating in gradient alcohol (75%, 85%, 95%, and 100%) and xylene (100%), then embedded in paraffin, and sectioned at 5 mm thickness, subsequently followed by staining in hematoxylin and eosin after euthanasia tissues staining. The sections were assessed by light microscopy (Olympus, Japan) under 100 × magnifications.

### 2.7. Statistical Analysis

All values were expressed as the mean ± standard deviation (SD). A minimum of three independent experiments was carried out for each assay. The statistical significance of data comparisons was determined using one-way analysis of variance (ANOVA). Values of p < 0.05 were considered statistically significant. Statistical analysis using SAS system software (version 9.1, SAS Institute, Cary, NC, USA) was used to calculate F values and compare between means by Duncan's multiple range test. Two statistical models were used to estimate phenotypic traits as follows:

(8a) Yijk=μ+Ai+Bj+ABij+eijk

(8b) Yijkl=μ+Ai+Bj+Ck+ABij+ACik+BCjk+ABCijk+eijkl

where Y_ijk_ is phenotype traits; *μ* is the overall mean; A_i_ is the effect of the i^th^ strains; B_j_ is the effect of the j^th^ time levels; C_k_ is the effect of the k^th^ concentrations or antibiotic types. Further, AB_ij_ is the interaction between i^th^ strains and j^th^ time levels. Moreover, AC_ik_ is the interaction between strains and k^th^ concentrations or antibiotic types, BC_jk_ is the interaction between j^th^ time and k^th^ concentrations or antibiotic types, ABC_ijk_ is the interaction between i^th^ strains, j^th^ time levels, and k^th^ concentrations or antibiotic types, and e_ijkl_ is the effect of the random error.

## 3. Results

### 3.1. PCR Amplification of the 16S rDNA and Sequence Analysis

**Figure 1:**
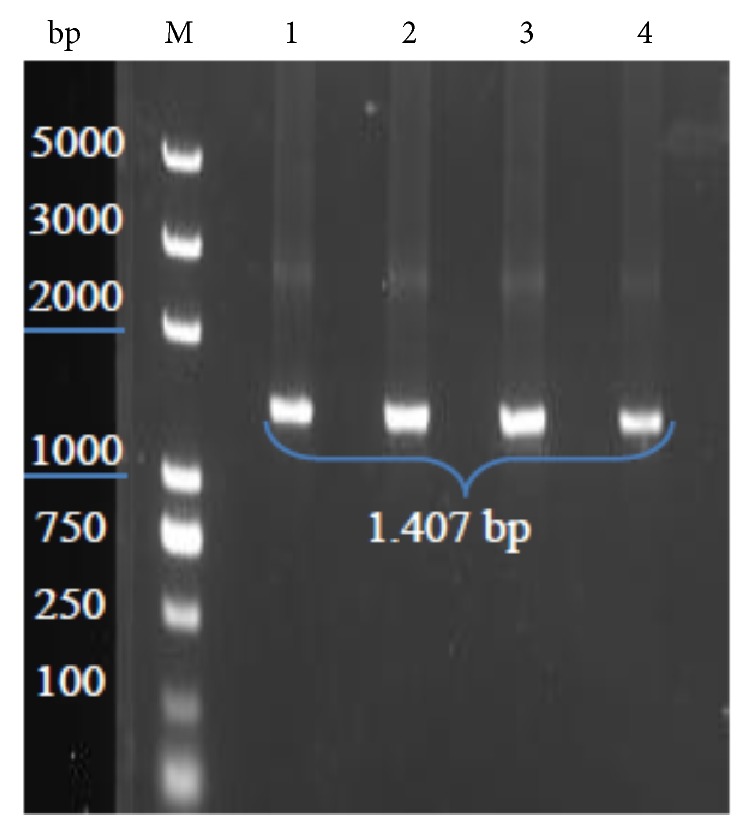
PCR amplification of* gad* gene. M: marker; 1, 2 PCR products of* Lactobacillus brevis* KLDS 1.0727 and 3, 4 of KLDS 1.0373.

Figure 1 showed the PCR amplification results of the presence* gad* gene and the nucleotide sequence of* gad* displayed the 1,407 bp. Further, our previous study reported the HPLC chromatogram analysis for GABA produced by KLDS 1.0727 and KLDS 1.0373 was 1.98±0.07 and 0.05±0.05 g/L, respectively (data no shown). Several studies have shown that the analysis of the sequence of nucleotide* gad* showed cloned genes consisting of 1,407 bp and 468 amino acids were encoded [31]. These results were closely related to the findings by Hiraga et al. [32] who suggested that the* gad* gene of* Lactobacillus brevis* IFO 12005 was 1,440 bp. Moreover, Wu et al. [19] reported that biochemical analysis and genetic screening have confirmed the common existence of* gad* system in* Lactobacillus brevis* suggesting its species-specific characteristic of GABA production.

### 3.2. Assessment of* Lactobacillus brevis* KLDS 1.0727 and KLDS 1.0373 In Vitro

The in vitro results obtained in three main Tables 2, 3, and 4 will be expressed to show the effect of some factors (strain types, time level, concentrations, and interaction of different parameters) as the equations of the statistics model mentioned above.

### 3.3. Determination of the Growth Rate and Bile Tolerance of* Lactobacillus brevis* KLDS 1.0727 and KLDS 1.0373 Strains

**Figure 2:**
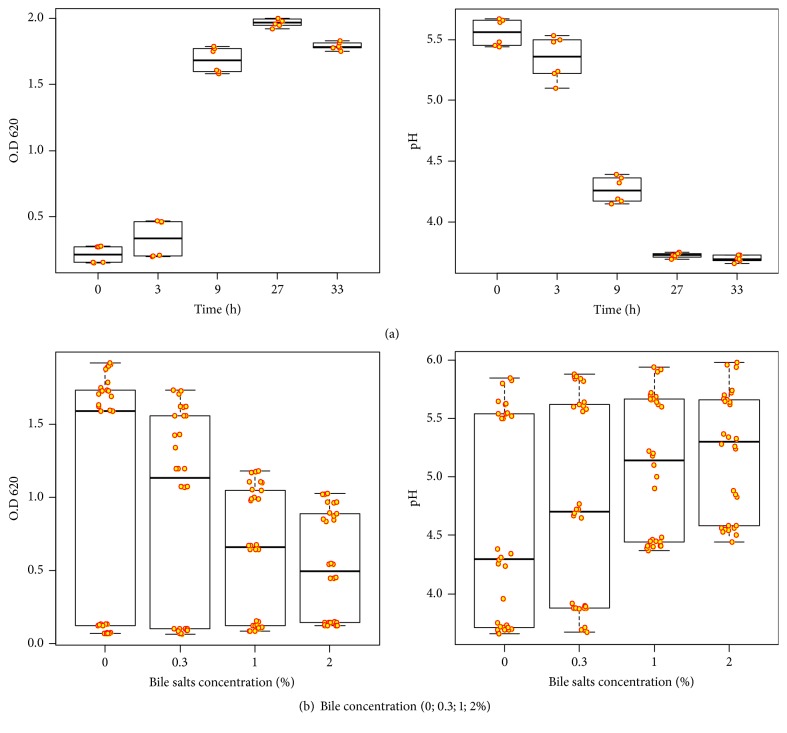
Determining a standard curve for growth rate/hours and tolerant bile (0; 0.3; 1, 2%) with accumulated acid of KLDS 1.0727 and KLDS 1.0373 strains. (a) Growth rate and pH; (b) bile salts and pH.

The obtained results in Figure 2 showed the standard curve of growth rate and bile tolerant with the accumulated acid of KLDS 1.0727 and KLDS 1.0373 strains. There was a significant positive correlation between growth rate and pH (0.98). Duncan comparisons between strains showed highly significant differences between KLDS 1.0727 and KLDS 1.0373 as shown in Table 2. KLDS 1.0727 showed a high growth rate and decreased pH values more than KLDS 1.0373 (1.24 ± 0.19 and 1.15 ± 0.21, respectively), where the pH is shown in Table 3 as follows (4.45 ± 0.20 and 4.59 ± 0.23, respectively). Meanwhile, the comparison between the time levels of the growth rate in Table 2 shows highly significant differences between all-time levels with the highest mean time at 27 hours (1.97 ± 0.01) and lowest at zero hours (0.22 ± 0.03) whereas pH at 33 hours was the highest values (3.70 ± 0.01) and the lowest values at zero hours (5.56 ± 0.05).

Meanwhile, Table 4 displayed the highest survival rate of strain KLDS 1.0373 at 3 hours and 0.3 % of bile salts (1.97±0.07^b^). Moreover, the lowest survival rate in KLDS 1.0727 after 9 hours and 2% of bile (1.78±0.07). Otherwise, the effect of bile salts concentration on growth rate and pH (P <0.001) was observed, and there was a significant correlation between the growth rate of strains and pH values (0.97). In addition, according to Table 2, there was a little significant difference between two strains. KLDS 1.0727 showed higher growth rate and pH values than KLDS 1.0373, 0.79 ± 0.08 and 0.76 ± 0.08; 4.90 ± 0.10 and 4.92 ± 0.10, respectively.

As Duncan's multiple range test mentioned above, the comparison between time levels of growth rate showed highly significant differences between all-time levels with the highest pH 1.40 and the lowest 1.10. Furthermore, Table 3 showed the highest pH 4.11 and the lowest 5.80. Table 2 showed the comparison between the concentration levels of the growth rate that explained high significant differences between all categories of bile concentration with the highest mean in zero % of the bile (1.08 ± 0.15) and was lower at 2% (0.52 ± 0.07) while pH with the highest values in zero% (4.60 ± 0.16) and lowest in 2% of bile (5.21 ± 0.10).

### 3.4. Resistance of KLDS 1.0727 and KLDS 1.0373 to Different Types of Antibiotics

**Figure 3:**
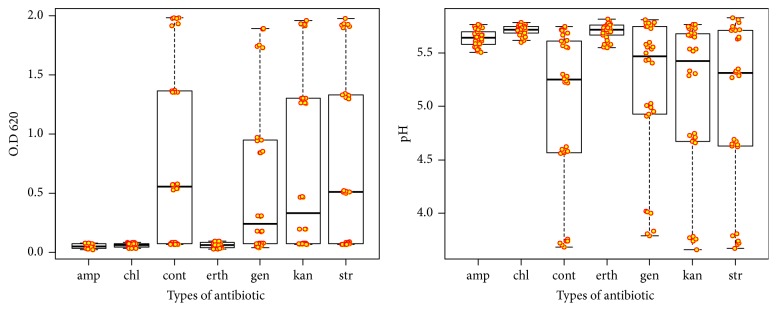
Resistance of KLDS 1.0727 and KLDS 1.0373 to different types of antibiotic as (OD)_620_ or pH. (amp), ampicillin; (chl), chloramphenicol; (cont), control; (erth), erythromycin; (gen), gentamicin; (kan), kanamycin; and (str), streptomycin.

Sensitivity to antibiotics is the most important factor in assessing the safety of probiotics and affecting the growth rate of strains and pH. Moreover, it is a potential threat to vital applications [33].  Figure 3 showed the effect of different types of antibiotics on the viability of* Lactobacillus brevis* strains. The obtained data in Table 2 referring to all factors and all possible interactions were highly significant (P<0.001). The correlation between bacterial growth rate and pH values (0.99) was highly significant as well. KLDS 1.0373 showed high growth rate and pH than KLDS 1.0727, 0.45 ± 0.06 and 0.44 ± 0.06 for growth rate; 5.33 ± 0.06 and 5.32 ± 0.06 for pH, respectively. Time levels showed significant differences between all-time categories with the highest growth rate at 24 hours (0.10 ± 0.15) and the lowest values at 3 hours (0.05 ± 0.003). Additionally, Table 3 showed the highest pH values at zero time (5.74 ± 0.01) and the lowest values at 24 hours (4.55 ± 0.14). Antibiotics showed highly significant differences between all antibiotics with the highest growth rate of control (0.80 ± 0.14) and the lowest in ampicillin (0.05 ± 0.003) and the same results for pH with the highest pH in chloramphenicol (5.71 ± 0.01) and the lowest in control (4.97 ± 0.14). It is expected since when LAB did not grow, there is no lactic acid production and vice versa.

Table 4 showed the factors affecting survival rate was significant as well as the interaction between the factors (P <0.0001). KLDS 1.0373 showed the highest survival at 3 hours of streptomycin (1.61 ± 0.01, 2.75 ± 0.02, and 2.36 ± 0.14, respectively). Meanwhile, erythromycin showed the lowest effect on strain KLDS 1.0727 at 6 hours (1.41 ± 0.09, 1.29 ± 0.14 and 0.83 ± 0.09 respectively). Notably, the sensitivity of KLDS 1.0727 after 24 hours to ampicillin, chloramphenicol, and erythromycin was 97.46, 97.91, and 98.11%, respectively. Further, KLDS 1.0373 was 98.81, 98.38, and 98.61%, respectively. On the other hand, the sensitivity of KLDS 1.0727 after 6 hours to streptomycin, gentamicin, and kanamycin was 12.18, 69.64, and 66.22%, respectively, and KLDS 1.0373 showed 2.28, 41.82, and 11.40, respectively.

### 3.5. Tolerance KLDS 1.0727 and KLDS 1.0373 to Simulated Intestinal Juice (SIJ) with Different pH under Aerobic or Anaerobic Conditions

**Figure 4:**
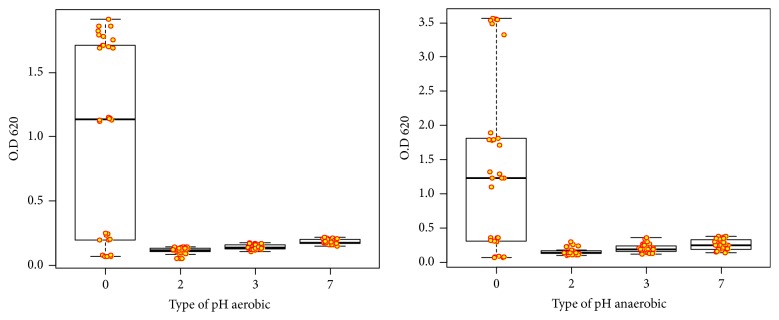
Tolerance of KLDS 1.0727 and KLDS 1.0373 to simulated intestinal juice (SIJ) with different pH under aerobic or anaerobic conditions.

The tolerance of GIT conditions is an important criterion for the selection of potential probiotics. Several studies have reported that MRS broth with pH value (2.0 - 3.0) was used to determine Lactobacillus acid resistance [34, 35].  Figure 4 shows the stander curve of the growth rate of KLDS 1.0727 and KLDS 1.0373 to tolerate (SIJ) with different levels of pH under aerobic or anaerobic condition. The data showed no significant effect (P < 0.05) growth rate of strains. However, the time and different pH and their interaction were extremely high (P <0.001). Table 2 indicates that the highest growth rate at 24 hours aerobically in control without any addition of (SIJ) was (0.56 ± 0.15 and 1.00 ± 0.14, respectively), and the lowest growth rate at zero time with pH 2.0 was (0.14 ± 0.01 and 0.12 ± 0.01, respectively). The obtained data in Table 4 showed that the growth rate and interaction between the factors were very significant (P <0.0001). The Duncan groups showed the highest survival rate for KLDS 1.0727 at zero time and pH 7.0 (1.16 ± 0.11; 2.08 ± 0.10, and 1.38 ± 0.16 respectively). Meanwhile, KLDS 1.0373 at pH 2.0 showed lower survival rate (1.13 ± 0.10; 0.56 ± 0.01 and 0.94 ± 0.09, respectively). These results were consistent with those obtained by de Almeida Júnior et al. [36]. On the other hand, Table 2 showed significant differences between strains and time under anaerobic condition with different factors (P <0.001) and the significant interaction between them (P <0.05) KLDS 1.0373 at 24 hours in pH control showed the highest growth rate (0.51 ± 0.10, 1.05 ± 0.29 and 1.39 ± 0.23, respectively). However, KLDS 1.0727 strain at zero time and pH 2.0 values were the lowest (0.49 ± 0.10, 0.14 ± 0.01 and 0.15 ± 0.01, respectively). Furthermore, Table 4 showed the survival rate was influenced by the same factors (P <0.0001). The highest survival rate of the KLDS 1.0373 at zero time and pH 7.0 was 1.17 ± 0.09, 2.12 ± 0.10 and 1.34 ± 0.14, respectively, while the survival rate of KLDS 1.0727 was the lowest at 24 hours and pH 2.0 (1.12 ± 0.10, 0.52 ± 0.02 and 0.96 ± 0.09, respectively).

### 3.6. Screening of the Antagonistic Properties of* Lactobacillus brevis* KLDS 1.0727 and KLDS 1.0373 Strains against Foodborne Pathogenic Bacteria

Due to the biological diversity functions of probiotic bacteria, there is a growing need for new strains of the LAB to play an important role in identifying dominant bacterial communities within intestinal ecosystem [35]. In this study, KLDS 1.0727 and KLDS 1.0373 have been tested against some foodborne pathogens, namely,* Salmonella typhimurium *ATCC 14028,* E. coli* IQCC 10126,* Listeria monocytogenes* IQCC 22221, and* Staphylococcus aureus* IQCC 22035.  Table 5 showed the strongest antimicrobial activity of KLDS 1.0727 and KLDS 1.0373 strains against all the indicator pathogens as expressed in terms of diameter of clear zones (mm). Therefore, the clear zone ranging from (1.93 ± 0.07 to 2.47 ± 0.03 mm) against all test indicators.

### 3.7. Caco-2 Cell Adhesion Assay

**Figure 5:**
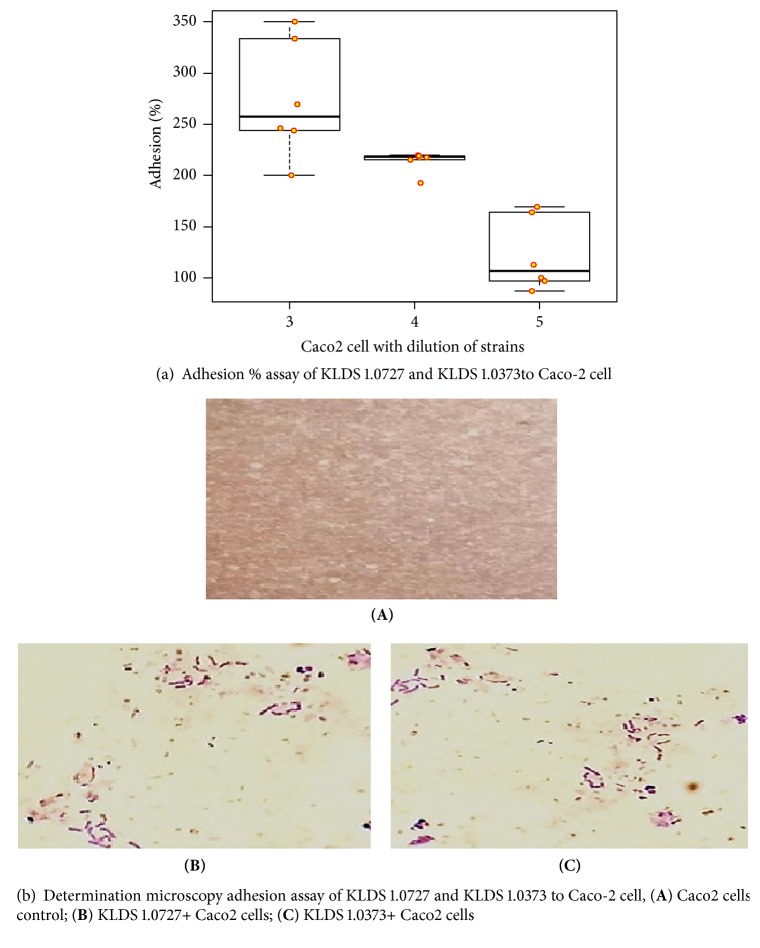


The most important considerations for the selection of probiotics are not only the ability to survive and transit through the digestive system but also the adhesion, establishment, or reproduction within the gastrointestinal tract. The obtained results in Figure 5(A) showed that the adhesion % of the KLDS 1.0727 and KLDS 1.0373 strains was (55.6 - 95.2%), therefore using three different dilutions KLDS 1.0727 and KLDS 1.0373 strains as 10^3^, 10^4^, and 10^5^. Moreover, Table 3 showed the effect of strain types on the adhesion % was nonsignificant (0.92), while the concentration effect was highly significant (p<0.0001). KLDS 1.0727 strain in the third dilution fold was the highest adhesion percentage (213 ± 0.19 and 268 ± 0.15, respectively), and KLDS 1.0373 strain in the dilution five was the lowest values (211 ± 0.20 and 148 ± 0.12 respectively). Moreover, in Figure 5(B) microscopic slides showed a high adherence % of KLDS 1.0727 and KLDS 1.0373 strains.

### 3.8. Intervention Effect of KLDS 1.0727 and KLDS 1.0373 Strains on Type I Diabetes In Vivo

#### 3.8.1. Hypoglycemic Activity and Body Weight of C57BL/6 Mice with Streptozotocin- (STZ-) Induced Diabetes during Four Weeks

**Figure 6:**
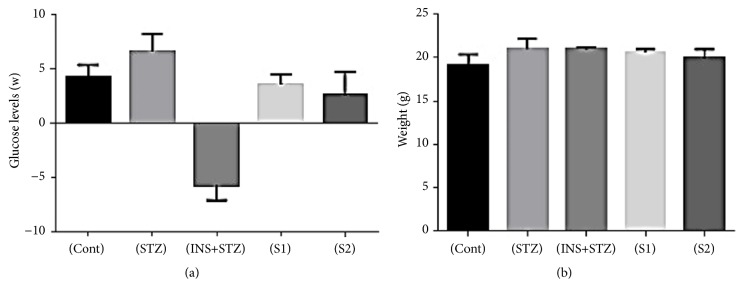
Blood glucose mmol/dl and body weight/g of C57BL/6 mice with streptozotocin- (STZ-) induced diabetes during four weeks. (a) Average of blood glucose level, (b) body weight/g. (Cont), control; (STZ), streptozotocin; (INS+STZ), insulin; (S1), KLDS 1.0727; (S2), KLDS 1.0373.

Glucose levels and body weight were checked weekly for five treated groups namely (Cont, STZ, STZ+INS; S1, and S2). The obtained results in Figure 6 showed a chronic increase in blood glucose level overnight fasting 12 hours and 2 hours postprandial and body weight within four weeks. The data presented the glucose levels as equation mentioned above for Cont; STZ; INS+STZ; S1; S2 was 4.2675±1.11; 6.595±1.63, -5.7375; 3.58±0.94, and 2.5925±2.15, respectively. In addition, the effect of STZ causing diabetes for C57BL/6 mice had a different effect on mice body weight. It was nonsignificant for all completely treated groups. Briefly, the lowest blood glucose and the highest body weight was in (INS+STZ) group (-5.7375; and 21.12 ± 2.54^a^ respectively). Notably, the (INS + STZ) group showed the blood glucose levels before eating or insulin injection were higher than the blood samples taken 2 hours postprandial. In contrast to all other groups, this may be due to insulin injection that promotes glucose uptake in blood.

### 3.9. Blood Plasma Assay

#### 3.9.1. Serum Biochemistry Parameters Assay

After overnight fasting, mice have been killed and blood serum was collected, and serum biochemistry parameters were measured.  Table 6 displayed the serum lipids concentrations as triglyceride (TG); total cholesterol (CHOL); high-density lipoprotein cholesterol (HDL); low-density lipoprotein cholesterol (LDL); glucose (GLU); magnesium (Mg^+2^) as well. All treated groups were highly significant, whereas Cont and STZ showed a high level of TG (2.54±0.91 and 2.06±0.84 mmol/L, respectively). Notably, all groups displayed a high level of Mg^+2^ and low level of LDL. Further, Cont group showed the highest level of CHOL (7.25±0.93^b^). On the other hand, INS+STZ showed the highest level of glucose (9.40±3.84^b^). Worthy of notice, S1 falls within the reference range of TG, CHOL, HDL, LDL, and GLU. Therefore, it can conclude that Cont showed the highest level of TG, CHOL, HDL, and LDL, while STZ showed the highest level of Mg^+2^, Moreover, INS+STZ showed the lowest level of CHOL and Mg^+2^ and the highest level of GLU. On the contrary, S1 expressed the lowest level of TG, LDL, and GLU.

### 3.10. Liver Functions Parameters

Liver functions parameters evaluated were the serum alanine aminotransferase (ALT); aspartate transaminase (AST); AST/ALT; total bile acid (TBA); albumin (ALB); globulin (GLUB); total protein (TP).  Table 6 showed (TP) total protein of all treated experiments falls in the average reference range except STZ that was (84.90±34.66). (ALT) serum alanine aminotransferase showed a dramatic increase in all treated groups and out of average reference range except (S2), (36.00±14.70). In addition, (AST) aspartate aminotransferase showed a significant increase in the levels of all treatment groups and also exceeded the mean of the reference range. Further, total bile acid (TBA) was expressed at a reasonable level for all treated groups, while albumin (ALB) and globulin (GLUB) display results are coherent for all treated groups where it falls in the average of the reference range. Briefly, STZ showed the highest level of TP, AST, ALB, and GLUB compared to all treated groups.

### 3.11. Kidney Function Parameter

Table 6 determined the kidney functions parameters such as uric nitrogen (BUN), creatinine (CREA), and uric acid (URIC) levels. The results displayed not alarming increase in uric nitrogen (BUN) and, further, creatinine (CREA) and uric acid (URIC) levels as well. All treated groups fall in the average of the reference range except Cont and S1 which expressed the lowest level of uric acid (URIC) (76.50±31.23^b^ and 125.60±51.28^ab^, respectively).

### 3.12. Insulin Blood Plasma Assay

Table 6 exhibited that all the treated groups fall within the reference range average. Therefore, Cont and S1 displayed the heights of groups in insulin levels as 13.52±0.23 and 11.13±0.28, respectively. Meanwhile, STZ and INS+STZ expressed the lowest levels as 9.99± 0.36 and 10.60±0.21, respectively.

### 3.13. Histological Evaluation

**Figure 7:**
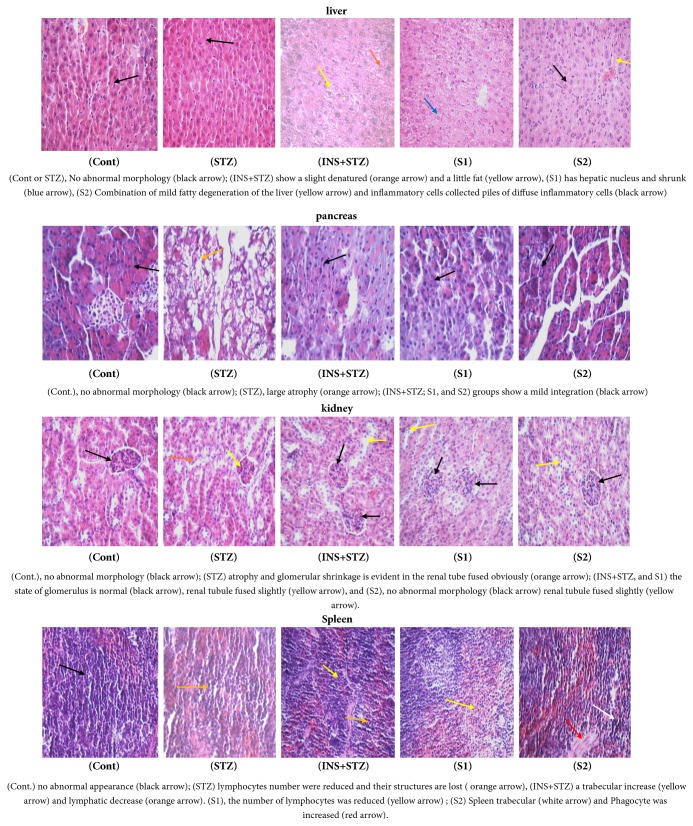
Histological examination of sacrifice mice organs (liver; pancreas; kidney, and spleen) streptozotocin-induced diabetes. (Cont), control; (STZ), streptozotocin; (INS+STZ), insulin; (S1), KLDS 1.0727, and (S2), KLDS 1.0373.

The slides in Figure 7 exhibited the histological mice organs (namely, liver; pancreas, kidney, and spleen). The figure illustrates that the livers histology of different groups as follows, no abnormal morphology in Cont or STZ groups, while INS+STZ showed a slight denatured and a little fat. Further, S1 group was hepatic nucleus and shrunk as well. S2 shows a combination of mild fatty degeneration of the liver and inflammatory cells collected piles of diffuse inflammatory cells. Moreover, the pancreas histology exhibited that there was no abnormal morphology in the Cont while STZ was a significant atrophy in pancreatic islets cells with a huge vacuolar degeneration. INS+STZ, S1, and S2 groups showed a mild integration. The kidneys histological slides displayed that Cont has no abnormal morphology, STZ group was atrophy, and glomerular shrinkage is evident in the renal tube fused obviously. Further, the state of INS+STZ and S1 glomerulus is normal, renal tubule fused slightly, and S2 has no abnormalities. Finally, the spleens histology exhibited that Cont has no abnormal appearance. Meanwhile, STZ group displayed lymphocytes number reduced and their structures are lost. Obviously, INS+STZ showed a trabecular increase and lymphatic decrease and the number of lymphocytes obviously reduced in S1, whereas S2 demonstrates that the spleen trabecular and phagocyte were increased.

## 4. Discussion

One of the key terms of probiotics is that they must withstand the harsh conditions of gastrointestinal juices, and, further, the ability to survive in the presence of bile, pancreatic juice, until the intestine is sufficient in number and provides health benefits for the host [37, 38]. In this study, two strains of* Lactobacillus brevis* were genetically determined ‎ and tested in vitro for some inappropriate simulated gastrointestinal conditions, such as acidity, bile tolerance, antibiotic resistance, and antimicrobial activity. Furthermore, in vivo experiments such as blood glucose; body weight; blood plasma; histological assay were tested to demonstrate the role of* Lactobacillus brevis* strains in the intervention of type 1 diabetes.

*Lactobacillus brevis* KLDS 1.0727 and KLDS 1.0373 is Gram-positive of lactic acid bacteria, which is a rod-type strain with high GABA generating capacity due to the* gad* gene. To confirm the identification of KLDS 1.0727 and KLDS 1.0373 strains, 16S rDNA was amplified and 540 bp of the DNA sequence was determined (unpublished data). The GenBank database was used to search for genes as the 16S rDNA sequence that revealed the highest similarity in the nucleotide chain was 99% with* L. brevis* (GenBank access no AF515220) and* L. brevis* (GenBank access no. AF515219). Therefore, the strains KLDS 1.0727 and KLDS 1.0373 are one of* L. brevis* strains.

The basic logistics model of SAS software suitable for experimental data showed that the factors (strains, time, and interaction between them) had significant effects on the growth rate of KLDS 1.0727 and KLDS 1.0373 strains and pH values (P <0.0001). Therefore, the obtained data were consistent with Liao et al. [39] who reported that pH values declined dramatically rapid in the first 9 hours and then slowly declined, attributed to strains that reached the stationary phase. In general, LAB growth reaches the stationary phase when the pH is less than 4.5. Zhang and Yew [40] reported that the acid resistance of LAB strains is dependent on specific strains and species.

*Lactobacillus brevis* strains were examined and tested for resistance and survival in acidic environment, as well as for growing in the presence of 0.3-2% bile salts; a similar concentration was found in the small intestine [41]. The obtained results showed* Lactobacillus brevis* strains were able to grow in high bile concentrations (2%). Similar results were previously reported by other researchers to study vital LAB strains from different environments [42]. The resistance of KLDS 1.0727 and KLDS 1.0373 strains of bile salts in MRS incubated at 37°C with 0.3% concentration of bile salts was closely related to bile level as reported by Sahadeva et al. [38]. In the gastrointestinal tract, observations were common between comparing different strains and different concentrations of bile, such as 0.3, 1, and 2%. These concentrations were closely associated with Hyacinta et al. [43] who explained the vitality of microorganisms affected by concentrations of bile salts.

Antibiotic is one of the most important growth determinants of most microbes and their resistance to growth is a criterion for selection of probiotic bacteria. Therefore, in the present study, the obtained data were in agreement with Klare et al. [44] who confirmed that LAB is usually sensitive to antibiotics such as chloramphenicol. de Almeida Júnior et al. [36] studied that 96% of disabling strains were sensitive to chloramphenicol. Furthermore, our findings were in agreement with Tulumoglu et al. [35] who reported that 90% of the tested Lactobacillus strains were resistant to gentamicin; meanwhile other studied strains were sensitive to ampicillin and erythromycin.

*Lactobacillus brevis* strains are a facultative anaerobic or microaerophilic LAB. Therefore, these explain our obtained results. Wu and Shah [45] concluded that ventilation has a bad effect on the feasibility and production of lactic acid and GABA by* Lactobacillus brevis*. The obtained results were consistent with those of Zhang [46], who showed the performance of antiacids of isolated strains. In addition, Wu et al. [19] reported that pancreatic fluid had no significant effect on survival of LAB.

Lactic acid bacteria have extremely antagonists against foodborne pathogenic bacteria [47]. Gautam and Sharma [48] report similar studies with* Lactobacillus spicheri* G2 showing 60% of hostility against different test indicators. Further, the obtained results were closely correlated with the findings of Fossi et al. [49]. A number of studies reported the potential antimicrobial mechanisms of specific microorganisms for the control pathogens are indicated as (i) production of antimicrobial compounds, (ii) production of bacteriocins, (iii) competitive action on nutrients, (iv) inhibition of binding due to competition, and (v) formation of the immune system [50]. Moreover, these findings agreed with our obtained results that show an extremely antagonism against common foodborne pathogenesis.

The adhesion of the intestinal mucosa is one of the most important features of probiotic bacteria to reproduction and produce biological compounds in the host digestive system [51]. LAB adhesion is a complex process involving the communication between the bacterial cell membrane and the interacting surfaces. Ehrmann et al. [52] reported that the ability of LAB to adhere to other cells is related to cell surface charge and hydrophobicity of bacteria. The ability to hold hydrocarbons can be expressed as cell surface hydrophobicity [53]. Moreover, Solieri et al. [34] reported that the hydrophobicity above 70% is considered highly hydrophobic. The obtained results were consistent with the study conducted by Zhang [46] who found that the highest hydrophobicity of the strains was 92.15 %. Our results showed that KLDS 1.0727 and KLDS 1.0373 have a similar adhesion value to the recognized probiotic strain* Lb. rhamnosus* GG, suggesting that it may be well-colonized in vivo.

Recently, diabetes has become the most common disease in the world. It causes many complications; hence high blood glucose levels may always damage tissues, especially capillaries and glomerular mesenchymal cells [54]. Streptozotocin (STZ) has been used clinically as a chemotherapeutic agent in the treatment of pancreatic cell *β*-cell carcinoma [55]. Reference ranges for blood tests are sets of values used by a health professional to interpret a set of medical test results from blood samples. In addition, examples of reference domains are given, varying by age, sex, health, and ethnicity, a method of analysis and measurement units. Individual results should always be interpreted using the reference range provided by the test laboratory.

Glucose serum (GLU) was reported by Wasserman [56]. The blood glucose test can measure the liver's ability to produce glucose, usually the last function to be lost if the liver fails. Glucose levels are usually the lowest in the morning before the first meal of the day and increase after meals for one to two hours. Blood sugar levels outside the normal range may be indicative of a medical condition. The higher level systematically refers to the high level of blood sugar. Low levels are known as hypoglycemia. Among the most observed cases, the obtained results are in close agreement with American Diabetes Association and Sacks et al. [57, 58] that suggest diabetes diagnosis historically included fasting blood glucose more than 7 mmol /dL (126 mg / dL) to 11.1 mmol/dL (200 mg / dL) or higher with hyperglycemia symptoms, or an abnormal oral glucose tolerance test of 2 hours.

High-density lipoprotein (HDL) is one of the five major groups of lipoproteins. HDL molecules are sometimes referred to as “good cholesterol” because it can transfer fat molecules from artery walls, reduce pharyngeal accumulation, and thus help to prevent or harden atherosclerosis. Therefore, the obtained results displayed that* Lactobacillus brevis* KLDS 1.0727 and KLDS 1.0373 strains have a slight similarity in the recommended ranges according to the American Heart Association, the National Institutes of Health, and the National Demining Program providing a set of guidelines on HDL levels and cardiovascular risk > 1.55 mmol / L [59].

Changes in ALT and AST levels are often used for liver pathological examination, AST /ALT is an important biochemical indicator [60]. ALT is mainly present in the liver cell plasma. When the liver cells are less damaged, the changes in membrane permeability of the liver cells will elevate the ALT blood levels. On the other hand, the increased levels of AST blood are the result of severe damage to liver cells. In other words, the higher AST / ALT ratio means more severe damage to liver cells. The findings results were in agreement with Prakasam et al. [61] who reported that the use of streptozotocin increases the level of AST, ALT, and ALP in blood plasma. Furthermore, the obtained results of KLDS 1.0727 and KLDS 1.0373 fall in the reference range and are in close similarity to total protein (TP) level 60.0-80.0 gm/dl. Besides, the total serum albumin (ALB) level is 35.0-55.0 g/Lb. Further, the total plasma globulin (GLUB) level is 25.0-40.0 g/Lb [62].

Renal dysfunction can be fatal for diabetic patients [63]. Hyperuricemia and hyperglycemia are the main causes of renal failure [63]. Diabetic nephropathy, high blood pressure, and high blood sugar are the main causes of renal failure. Kidney function in the kidney disease is an indicator of the activity, vitality, and overall health of kidney. The obtained results refer to the creatinine levels of all treated groups falling in the range of safe creatinine levels that is one of the most important kidney function tests and the results of this parameter are used to evaluate kidney functions [64].

Insulin is an anabolic hormone that promotes glucose uptake, glycogenesis, lipogenesis, and protein synthesis of skeletal muscle and fat tissue through the tyrosine kinase receptor pathway. In addition, insulin is the most important factor in the regulation of plasma glucose homeostasis, as it counteracts glucagon and other catabolic hormones epinephrine, glucocorticoid, and growth hormone. The obtained results are closely similar to Graham et al. [65] who indicated that the insulin range in blood plasma falls between 3 and 19 uIU/ml. Finally, based on previous studies, the role of probiotics in the development of T1D demonstrated that oral probiotics inhibit the development of T1D in diabetic mice model. Therefore, probiotics showed the ability to the intervention of diabetes [15].

## 5. Conclusions

*Lactobacillus brevis* KLDS 1.0727 and KLDS 1.0373 strains were exhibited to be ‎extremely viable in in vitro experiments and exhibited survival in in vitro digestion models mimicking the gastrointestinal conditions. ‎Moreover, it shows significant hostility against foodborne pathogenic bacteria. In addition, ‎KLDS 1.0727 and KLDS 1.0373 strains expressed adequate GABA that demonstrate a ‎superb physiological effect in in vivo experiment. On the other hand, it shows a significant ‎effect in lowering blood glucose levels or insulin in plasma. In addition, most ‎functional liver and kidney tests fall within the reference range of plasma ‎blood parameters. Therefore, we recommended using* Lactobacillus brevis* KLDS ‎‎1.0727 and KLDS 1.0373 strains widely in the market as a natural source of GABA ‎in pharmaceutical and food applications to reduce the threat of type 1 diabetes.‎

## Figures and Tables

**Table 1:** Experiments design of diabetic model mice.

**Group Treatment**		**Dose/ mouse**	**Time of treated**	**Methods of delivered**
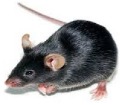	(Cont)	250 *μ*L Saline	Daily	via gavage
	(STZ)	180 mg/kg	One time	subcutaneously
	(INS+STZ)	100 *μ*L	Daily	subcutaneously
	(S1)	250 *μ*L	Daily	via gavage
Male C57BL/6	(S2)	250 *μ*L	Daily	via gavage

(Cont), control group; (STZ), streptozotocin; (INS+STZ), insulin + streptozotocin; (S1), KLDS1.0727+ streptozotocin, and (S2), KLDS1.0373+ streptozotocin.

**Table 2:** Significance, means, and standard errors (SE) of factors affecting the growth rate of KLDS 1.0727 and KLDS 1.0373 strains.

**SOV/Experiments**	**Growth rate**		**Bile tolerance**		** SIJ with different pH aerobically **		** SIJ with different pH anaerobically **		**Antibiotic resistance**		**Adhesion to Caco2 cell**	
	High	Low	High	Low	High	Low	High	Low	High	Low	High	Low
**Strain**	(S1)	(S2)	(S1)	(S2)	(S1)	(S2)	(S2)	(S1)	(S2)	(S1)	(S2)	(S1)
**Mean±SE of strains**	1.24±0.19^c^	1.15±0.21	0.79 ±0.08^c^	0.76±0.08	0.36±0.07^d^	0.36±0.07	0.51±0.10^b^	0.49±0.10	0.45±0.06^c^	0.44±0.06	245 ±36.51^d^	244.67±33.31
**Time**	27	0	27	0	24	0	24	0	24	3	-	-
**Mean±SE of time**	1.97±0.01^c^	0.22±0.03	1.40±0.07^c^	1.10±0.01	0.56±0.14^c^	0.14±0.01	1.05±0.29^c^	0.14±0.01	1.11±0.15^c^	0.05±0.003	-	-
**Concentration of different parameters **	-	-	0	2	0	2	0	2	Cont.	Amp.	3	0
**Mean±SE**	-	-	1.08^c^ ±0.15	0.52±0.07	1.00±0.14^c^	0.12±0.01	1.39±0.23^c^	0.15±0.01	0.80 ±0.14^c^	0.05±0.003	381.67±48.47^c^	137.50±6.26

**R** ^**2 **^ *∗*	99.99		99.96		99.98		99.88		99.99		72.36	
**CV ** *∗*	0.67		2.11		2.64		6.71		1.83		30.48	

In the six experiments, **growth rate**: the effect of two strains KLDS 1.0727 (S1) and KLDS 1.0373 (S2), and five levels of times 0, 3, 9, 27, and 33h on growth rate and pH; **bile tolerance**: the effect of two strains, five levels of times, and four levels of bile salts concentration Cont, 0.3, 1, and 2% on growth rate and pH; **simulated intestinal juice (SIJ) with different pH aerobically**: the effect of two strains, five levels of times 0, 3, 6, 9, and 24, and simulated intestinal juice with different four levels of pH Cont, 2, 3, and 7 on growth rate; **simulated intestinal juice(SIJ) with different pH anaerobically**: the effect of two strains, five levels of times 0, 3, 6, 9, and 24, and simulated intestinal juice with different four levels of pH Cont, 2, 3, and 7; **antibiotic resistance**: the effect of two strains, five levels of times, and types of antibiotics Cont, streptomycin, ampicillin, chloramphenicol, erythromycin, gentamycin, and kanamycin on growth rate and pH; **adhesion with Caco2 cell**: the effect of two strains, four levels of dilution of 2 strains Cont, 3, 4, and 5 adhesion of Caco 2 cell. Significance: a, P < 0,01; b: P< 0,001; c: P< 0,0001; d: nonsignificant. *∗* R^2^: determination coefficient, and CV: coefficient of variation. *∗*SOV: source of variance.

**Table 3:** Significance, means, and standard errors (SE) of factors affecting pH of KLDS 1.0727 and KLDS 1.0373 strains.

**∗** **SOV/Experiments**	**Growth rate and pH**		**Bile tolerance**		**Antibiotics tolerance**	
	**High**	**Low**	**High**	**Low**	**High**	**Low**
**Strain**	(S2)	(S1)	(S2)	(S1)	(S2)	(S1)
**Mean±SE of strains**	4.59±0.23^c^	4.45±0.20	4.92±0.10^a^	4.90±0.10	5.33^b^ ±0.06	5.32±0.06
**Time**	0	33	0	33	0	24
**Mean±SE of time**	5.56 ±0.05^c^	3.70±0.01	5.81±0.02^c^	4.11±0.07	5.74^c^ ±0.01	4.55±0.14
**Concentration of bile or type of antibiotic**	-	-	2	0	Chloramphenicol	Control
**Mean±SE**	-	-	5.21 ±0.10^c^	4.60±0.16	5.72^c^ ±0.01	4.97±0.14

**R2**	99.96		99.85		99.92	
**CV**	0.5		0.73		0.4	

See footnote of Table 2.

**Table 4:** Significance, means, and standard errors (SE) of factors affecting survival or adhesion % of KLDS 1.0727 and KLDS 1.0373 strains.

**∗** **SOV/Experiments**	**Bile tolerance**		**(SIJ) with different pH aerobically**		**(SIJ) with different pH anaerobically**		**Antibiotic resistance**		**Adhesion to Caco2 cell**	
	**High**	**Low**	**High**	**Low**	**High**	**Low**	**High**	**Low**	**High**	**Low**
**Strain**	(S2)	(S1)	(S1)	(S2)	(S2)	(S1)	(S2)	(S1)	(S1)	(S2)
**Mean±SE of strains**	1.97±0.07^b^	1.78±0.07	1.16±0.11^b^	1.13±0.10	1.17±0.09^c^	1.12±0.1	1.60±0.10^c^	1.41±0.09	213±0.19^d^	211±0.20
**Time**	3	9	0	24	0	24	0	6		
**Mean±SE of time**	2.10±0.14^c^	1.52±0.09	2.08±0.10^c^	0.56±0.01	2.12±0.10^c^	0.52±0.02	2.75±0.02^c^	1.29±0.14		
**Concentration of bile or type of antibiotic**	0.3	2	7	2	7	2	Str.	Erth.	3	5
**Mean±SE of concentration**	2.17±0.09^c^	1.66±0.09	1.38±0.16^c^	0.94±0.09	1.34±0.14^c^	0.96±0.09	2.36±0.14^c^	0.83±0.09	268±0.15^c^	148±0.12

**R2**	86.6		99.71		99.64		96.48		0.78	
**CV**	12.04		3.87		3.94		13.91		15.2	

See footnote of Table 2.

**Table 5:** Antibacterial activity of the KLDS 1.0727 and KLDS 1.0373 strains against foodborne pathogenic bacteria in terms of ZDI.

**Test /foodborne strains**	**ZDI (mm) ± SD of Indicator Bacteria**							
	***Salmonella typhimurium***		***E. coli***		***Listeria monocytogenes***		***Staphylococcus aureus***	
**KLDS 1.0727**	2.37±0.03	+++	1.97± 0.03	+++	2.17±0.09	+++	2.20± 0.10	+++
**KLDS 1.0373**	2.47±0.03	+++	1.93±0.07	+++	1.93±0.07	+++	2.20± 0.10	+++
**Sig.**	<.0001		0.4226		0.0198		0.0572	
**R** ^**2**^	1.000000		0.904762		0.978495		0.970588	

+++ refers to clear zone (14 mm, 3 points; 9–14 mm, 2 points; 1–9.9 mm, one point).

**Table 6:** Blood plasma assay (n=4, mean±SE).

	**Ref. ranges**	**(Cont)**	**(STZ)**	**(INS+STZ)**	**(S1)**	**(S2)**
	**Plasma biochemical parameter**					
**TG mmol/** **L** **b** ^**∗∗∗**^	0.45 -1.7	2.54±0.91	2.06±0.84	1.02±0.42	0.96±0.39	1.34±0.55
**CHOL ** **mmol/** **L** ^**∗∗∗**^	2.85 – 5.7	7.25±0.93^b^	4.89±2.00^bc^	3.38±1.38^c^	3.46±1.41^c^	3.44±1.40^c^
**HDL ** **mmol/** **L** ^**∗**^	0.93 – 1.81	4.20±0.90^a^	1.55±0.63^b^	2.26±0.92^b^	2.39±0.98^b^	2.22±0.91^b^
**LDL ** **mmol/** **L** ^**ns**^	2.07 – 3.63	1.14±0.47^a^	0.83±0.34^ab^	0.49±0.20^b^	0.38±0.16^b^	0.48±0.20^b^
**Mg** ^**+2**^ ** mmol/** **L** **b** ^**∗**^	0.7-1.1	1.50±0.08	1.75±0.71	1.48±0.60	1.64±0.67	1.64±0.67
**GLU ** **mmol/** **L** ^**∗∗**^	3.57 – 6.12	4.4±1.27^bc^	4.40±1.80^a^	9.4±2.71^a^	3.1±0.89^c^	5.1±1.47^bc^

**Liver functions parameters**						

**TP** ** g/L** **b** ^**∗∗∗**^	60.0-80.0	60.30±0.90	84.90±34.66	58.10±23.72	60.90±24.86	64.90±26.50
**ALT ** **IU/L** **b** ^**∗∗∗**^	1.0 - 40.0	128.00±0.82	95.00±38.78	77.00±31.44	83.00±33.88	36.00±14.70
**AST IU/L** **b** ^**∗∗∗**^	1.0 - 40.0	263.00±0.82	341.00±139.21	176.00±71.85	274.00±111.86	182.00±74.30
**AST/** **A** **L** **T** ^**∗**^		2.05±0.01^b^	3.59±0.01^ab^	2.29±0.01^b^	3.30±1.35^ab^	2.89±1.18^b^
**TBA** ** mol/** **L** **b** ^**∗∗∗**^	0.01-20.0	8.50±0.90	4.90±2.00	3.90±1.59	2.60±1.06	3.90±1.59
**ALB ** **g/L** **b** ^**ns**^	35.0-55.0	34.50±0.90^a^	39.10±15.96^ab^	33.70±13.76^ab^	36.50±14.90^ab^	37.50±15.31^ab^
**GLUB ** **g/L** **b** ^**∗∗∗**^	25.0-40.0	25.80±0.90	45.80±18.70	24.40±9.96	24.40±9.96	27.40±11.19
**A/** **G** ^**ns**^	1.5 -2.5	1.34±0.01^a^	0.85±0.01^ab^	1.38±0.01^a^	1.50±0.61^a^	1.37±0.56^a^

**Kidney function parameter**						

**BUN ** **mmol/L** **b** ^**∗∗∗**^	1.07 – 7.14	8.60±3.51	10.50±4.29	8.00±3.27	11.80±4.82	10.80±4.41
**CREA** ** mol/L** **b** ^**∗∗∗**^	53.0 – 132.0	44.30±18.09	40.50±16.53	39.10±15.96	37.40±15.27	41.90±17.11
**URIC ** **Umol/** **L** ^**ns**^	142.0 –401.0	76.50±31.23^b^	175.10±71.48^ab^	180.60±73.73^ab^	125.60±51.28^ab^	164.90±67.32^ab^

**Insulin blood plasma**						

**Insulin ** **uIU/ml**	3-19	13.52±0.23	9.99± 0.36	10.60±0.21	11.13±0.28	10.93±0.20

TG, triglyceride; CHOL, total cholesterol; HDL, high-density lipoprotein cholesterol; LDL, low-density lipoprotein cholesterol; GLU, glucose; Mg^+2^, magnesium; ALT, serum alanine aminotransferase; AST, aspartate aminotransferase; AST/ALT, presence of aspartate transaminase and serum alanine aminotransferase; TBA, total bile acid; ALB, albumin; GLUB, globulin; TP, total protein; BUN, uric nitrogen; CREA, creatinine; URIC, uric acid. (Cont), control; (STZ), streptozotocin; (INS+STZ), insulin; (S1), KLDS 1.0727; (S2), KLDS 1.0373.

## Data Availability

The data used to support the findings of this study are available from the corresponding author upon request.
